# Ammonia Uptake from Ambient Air by Protonic Layered Metal Oxide as a Cause of the Gradual Degradation of Its Swelling Reactivity

**DOI:** 10.1021/acs.langmuir.5c03503

**Published:** 2025-08-29

**Authors:** Nobuyuki Sakai, Ritesh Uppuluri, Nobuo Iyi, Yasuo Ebina, Renzhi Ma, Takayoshi Sasaki

**Affiliations:** Research Center for Materials Nanoarchitectonics (MANA), 52747 National Institute for Materials Science (NIMS), 1-1 Namiki, Tsukuba, Ibaraki 305-0044, Japan

## Abstract

Protonic layered transition metal oxides readily undergo significant swelling and exfoliation into unilamellar nanosheets upon contact with aqueous solutions of amines and other basic compounds. Despite the crucial importance of this reactivity, it often degrades over time, leading to a loss of swelling and exfoliation capabilities. However, the cause of this degradation has remained unclear. In this study, we systematically investigated the conditions that lead to the loss of swelling ability, using protonic layered perovskite crystals of HCa_2_Nb_3_O_10_·1.5H_2_O as a representative example. The crystals were stored under various conditions, including ambient air, sealed environments, and dry/humid conditions, both in the dark and under light. Their reactivity with dimethylaminoethanol (DMAE) was then examined. We found that only the crystals exposed to ambient air for more than 10 days lost their swelling ability, whereas those stored under other conditions retained their reactivity. Based on these results, we speculated that ammonia, the most abundant alkaline component in the atmosphere, is absorbed from the air via an acid–base interaction. This hypothesis was confirmed through XRD and FTIR characterization. We conclude that trace amounts of ammonia (0.02–0.12 ppm) in the atmosphere intercalate into the acidic interlayer galleries and are responsible for the degradation in reactivity. Furthermore, the original reactivity can be easily restored by acid treatment, which replaces the intercalated ammonium ions with protons, thereby recovering the swelling capability. These findings resolve a long-standing question regarding the degradation of protonic layered metal oxides and provide a practical method for restoring their native properties.

## Graphical abstract

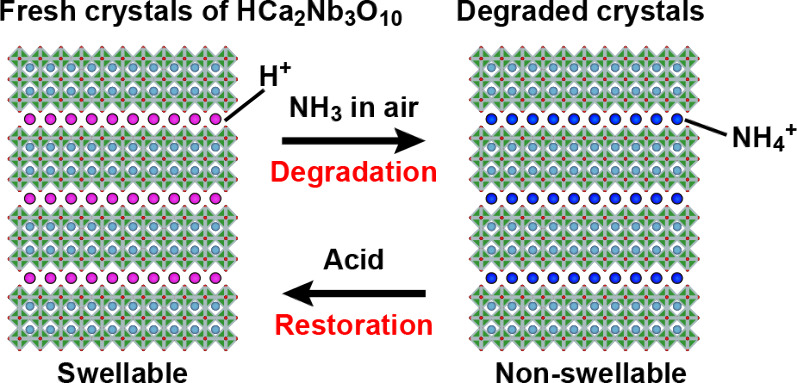

## Introduction

Layered transition metal oxides, composed of negatively charged host layers and interlayer cations, exhibit rich compositional and structural diversity. They have been extensively studied for a wide range of applications, including energy conversion and storage, magnetic materials and devices, sensing technologies, and catalytic systems.^1^ These compounds display ion-exchange properties under ambient conditions. Protonated layered oxides, obtained via acid exchange from precursor layered oxides, exhibit Brønsted acidity, enabling the intercalation of a variety of guest species, such as ions, molecules, and metal complexes, into the interlayer spaces. A wide range of nanocomposite materials useful in various applications have been fabricated through such solution-based processes.^2–5^ Recently, the reactivity of these materials has gained increasing attention due to the successful exfoliation of layered oxides driven by their solid-acid behavior. In particular, treatment with amines or organoammonium ions can induce significant swelling, ultimately leading to exfoliation into individual layers.^6,7^ The resulting molecularly thin, two-dimensional nanosheets have attracted considerable interest because of their novel and enhanced physicochemical properties.^8–15^

A key prerequisite for delaminating layered oxides into monolayer nanosheets is the osmotic swelling of the material, which expands the interlayer galleries through the absorption of water and other polar solvents containing amines or organoammonium cations.^16^ The osmotic swelling behavior depends primarily on the concentration of the amines and is largely independent of their type, whether primary amines, tertiary amines, or quaternary ammonium hydroxides, typically resulting in a 20–100-fold expansion of the interlayer spacing.^17–19^ However, it has been observed that the reactivity of protonated layered oxides gradually decreases over time, leading to a decline in their swelling capability. In our experience, it has long been believed that these materials are unstable under low-humidity conditions and/or higher temperatures and that deterioration is caused by drying or heating of the samples. Nevertheless, the true cause of this degradation has yet to be fully elucidated.

In this study, we systematically examined the degradation behavior of a protonated layered oxide by focusing on the swelling capability of HCa_2_Nb_3_O_10_·1.5H_2_O, which is known to swell and exfoliate in the presence of various alkylamines.^6,20–22^ We found that during storage in ambient air, the protonated layered compound gradually transforms into its ammonium ion-intercalated form, starting from the *c*-plane edges of the crystallites, resulting in a reduction in its swelling capability. The likely source of the ammonium is trace amounts of ammonia (0.02–0.12 ppm) present in indoor ambient air, originating from concrete walls and the human body.^23,24^ The swelling capability was successfully restored by acid treatment of the degraded compounds.

## Experimental Section

### Flux-Mediated Growth of KCa_2_Nb_3_O_10_ Microcrystals and Ion Exchange

Layered perovskite oxide KCa_2_Nb_3_O_10_ was synthesized via a flux-mediated growth method, as previously reported.^20,21^ Briefly, K_2_SO_4_, CaCO_3_, and Nb_2_O_5_ were mixed in a molar ratio of 5:4:3, ground using an agate mortar and pestle, heated in a platinum crucible at 1300 °C for 24 h, and then slowly cooled. In this process, K_2_SO_4_ served both as the potassium source and as the flux. After dissolving the flux in water, platy crystals with well-developed *c*-planes, measuring 25–125 μm in width and 5–10 μm in thickness, were collected by sieving and used in subsequent experiments. The KCa_2_Nb_3_O_10_ microcrystals were stirred in 5 mol dm^–3^ HNO_3_ for 3 days to convert them into the protonated form, HCa_2_Nb_3_O_10_·1.5H_2_O.^20,25^ The platy morphology and dimensions of the crystals remained largely unchanged after protonation.

### Test of the Swelling Behavior

Crystals of HCa_2_Nb_3_O_10_·1.5H_2_O were placed on a glass slide and exposed to various environmental conditions to examine their effects on swelling properties. The crystals were then treated with a 0.183 mol dm^–3^ solution of dimethylaminoethanol (DMAE) by applying a few drops of the solution directly onto the crystals on the slide. Changes in size and morphology were observed by using an Olympus BX51 optical microscope.

### Characterization

Powder X-ray diffraction (PXRD) data were collected by using a Rigaku RINT1200 diffractometer with Ni-filtered Cu Kα radiation (λ = 0.15418 nm). To examine the dependence of the basal spacing on the relative humidity (RH), measurements were also conducted by using a Rigaku RINT2000 diffractometer equipped with an RH-controlled chamber. Data were acquired at controlled RH levels ranging from 10% to 90% at 25 °C. Each measurement was initiated after maintaining the target RH for approximately 80 min. XRD analysis of a single crystal was performed using a Rigaku Saturn CCD single-crystal diffractometer equipped with VariMax confocal optics for Mo Kα radiation (λ = 0.071073 nm) at 20 °C and RH between 60% and 70%. Synthetic precession patterns were calculated and visualized by using the CrysAlis Pro software suite. Attenuated total reflection Fourier transform infrared (ATR-FTIR) spectra were recorded by using a PerkinElmer Spectrum One FTIR spectrometer equipped with a single-reflection ATR accessory (PerkinElmer L1200361, diamond/ZnSe top plate). Scanning electron microscopy (SEM) images were obtained using a JEOL JSM-6010LA instrument.

## Results and Discussion

The crystals of HCa_2_Nb_3_O_10_·1.5H_2_O used in this study exhibited a well-defined platelet morphology with lateral dimensions of 25–125 μm and exhibited sharp facets (Figure S1). Fresh crystals readily underwent swelling upon immersion in an aqueous DMAE solution, as evidenced by the significant elongation observed under a polarized optical microscope (Figure 1a,b). This osmotic swelling results from the exchange of protons in the crystal with DMAE, which is accompanied by water influx. In contrast, crystals that had been exposed to indoor ambient air in a laboratory for 11 days showed no swelling (Figure 1c). The loss of swelling capability may be attributed to environmental effects, such as excessive drying leading to the loss of interlayer water, photocatalytic reactions triggered by light exposure, or reactions with airborne gases.

**Figure 1:**
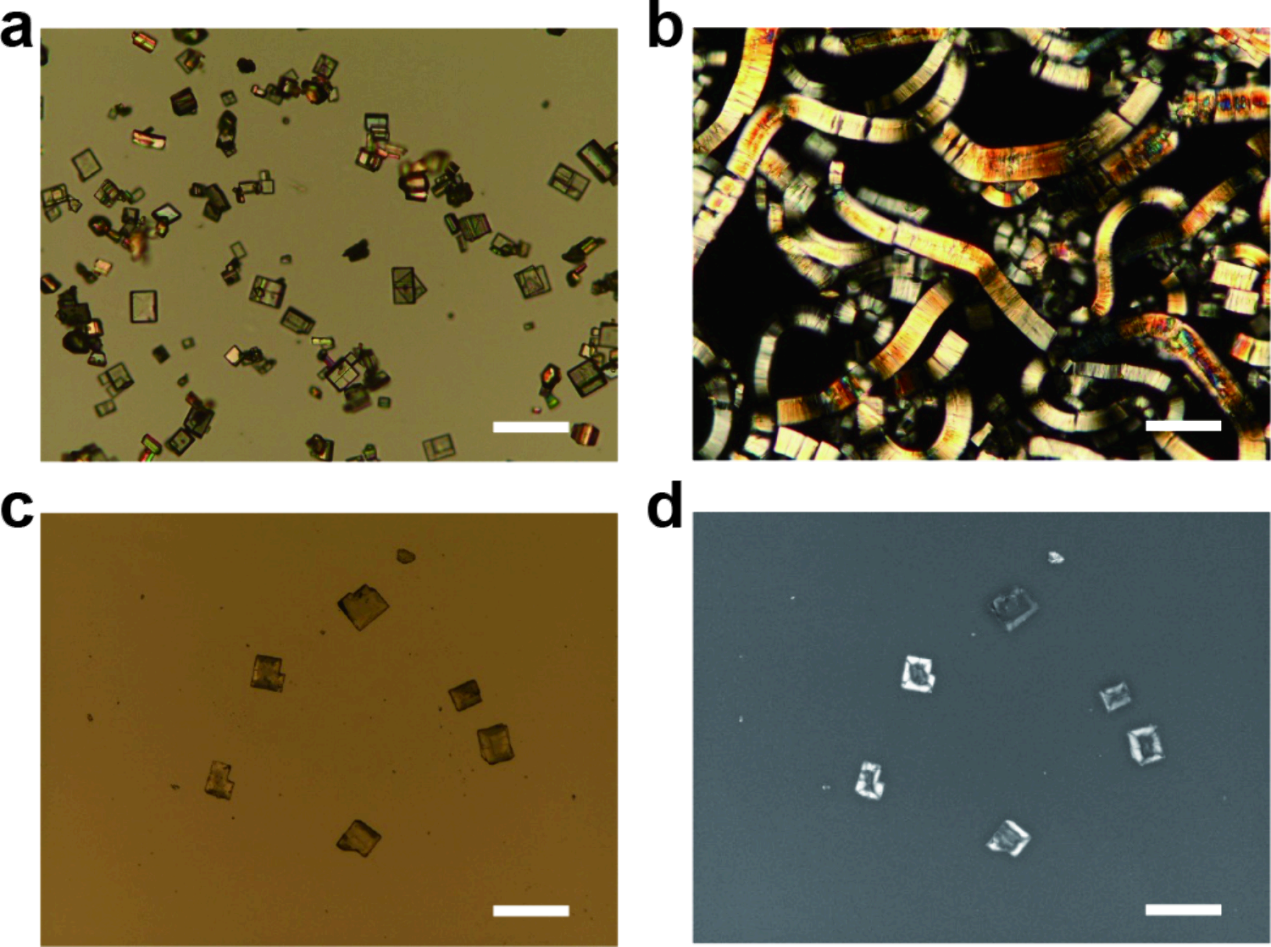
(a and c) Normal and (b and d) polarized optical microscopy images of (a and b) fresh and (c and d) degraded HCa_2_Nb_3_O_10_·1.5H_2_O crystals. Images were taken (a, c, and d) before and (b) after immersion in an aqueous DMAE solution. Scale bars represent 200 μm.

To identify the cause of degradation, HCa_2_Nb_3_O_10_·1.5H_2_O crystals were stored under various indoor environmental conditions, including (i) in a closed space to isolate them from the ambient environment; (ii) in open air, in the presence and absence of light, and (iii) in a desiccator with controlled RH levels of 25% and 70%, for a period of two months or longer. The swelling capabilities of the crystals after storage under these different conditions are summarized in Table 1. Crystals stored in sealed containers filled with ambient air, such as a 100 mL plastic box or a tightly capped 10 mL bottle, retained their swelling capability even after seven months, indicating that simple aging is not responsible for degradation. In contrast, crystals exposed to open ambient air lost their swelling ability regardless of whether they were kept in the dark or under fluorescent light, suggesting that photocatalytic degradation due to UV irradiation is not the cause. Crystals stored in an 11 L desiccator at 70% or 25% RH also retained their swelling capability. The preservation of swelling behavior even at 25% RH, where dehydration can occur, suggests that the loss of interlayer water is not a critical factor. In summary, the swelling capability is preserved when the crystals are stored in enclosed spaces filled with ambient air, whereas degradation occurs when the crystals are continuously exposed to the open atmosphere over extended periods. These results suggest that a trace component in air plays a key role in the loss of swelling capability.

**Table 1:** Swelling Capability of HCa_2_Nb_3_O_10_·1.5H_2_O Crystals Stored under Different Environmental Conditions

storage environment	duration	swelling capability
in a sealed plastic box (100 mL)	two months	swellable
in a tightly capped bottle (10 mL)	seven months	swellable
open atmosphere in the dark	two months	nonswellable
open atmosphere under light	two months	nonswellable
in a desiccator (11 L, 70% RH)	two months	swellable
in a desiccator (11 L, 25% RH)	two months	swellable

We found that the nonswellable crystals exhibited a “photoframe” texture,^26,27^ characterized by bright regions appearing at the periphery of the platy crystals under a polarized optical microscope (Figure 1d). This texture arises from differences in refractive index, likely caused by compositional changes near the crystal edges. The “photoframe” texture was not observed in crystals exposed to air for up to 3 days (Figure S2). However, some crystals stored for 7 days began to show this texture, and all crystals exposed to air for 14 days exhibited it. With continued exposure, the bright peripheral regions became thicker, and after 49 days, the bright regions had extended further, resulting in crystals that were entirely occupied by bright regions, particularly in smaller crystals. These results indicate that degradation or the loss of swelling capability proceeds gradually from the edges toward the interior of the crystals during storage in open ambient air.

Since the interlayer components of layered crystals are closely related to their hydration behavior, both fresh and degraded crystals were examined under various RH conditions. The crystals were first equilibrated in a dry atmosphere by being stored at 10% RH for 4 h, after which the RH was increased stepwise in 10% increments. After holding under each RH condition for an appropriate period (0.5–1 h), XRD data were recorded. The fresh crystals showed no change in the basal spacing (*d* = 1.44 nm) up to 50% RH, but at higher RH levels, an expansion of 0.17 nm was observed, resulting in the formation of a fully hydrated phase, HCa_2_Nb_3_O_10_·1.5H_2_O (*d* = 1.61 nm) (Figure 2a).^25^ In contrast, the degraded crystals that had been exposed to air exhibited different hydration behaviors in response to RH changes (Figure 2b). At <50% RH, a mixture of the 1.51 nm phase and the dehydrated 1.44 nm phase was observed. At >50% RH, the dehydrated phase transformed into the fully hydrated form (1.61 nm) whereas the 1.51 nm phase remained unchanged. These results indicate that the degraded crystals contain a nonhydratable phase, suggesting a change in the interlayer cation composition.

**Figure 2:**
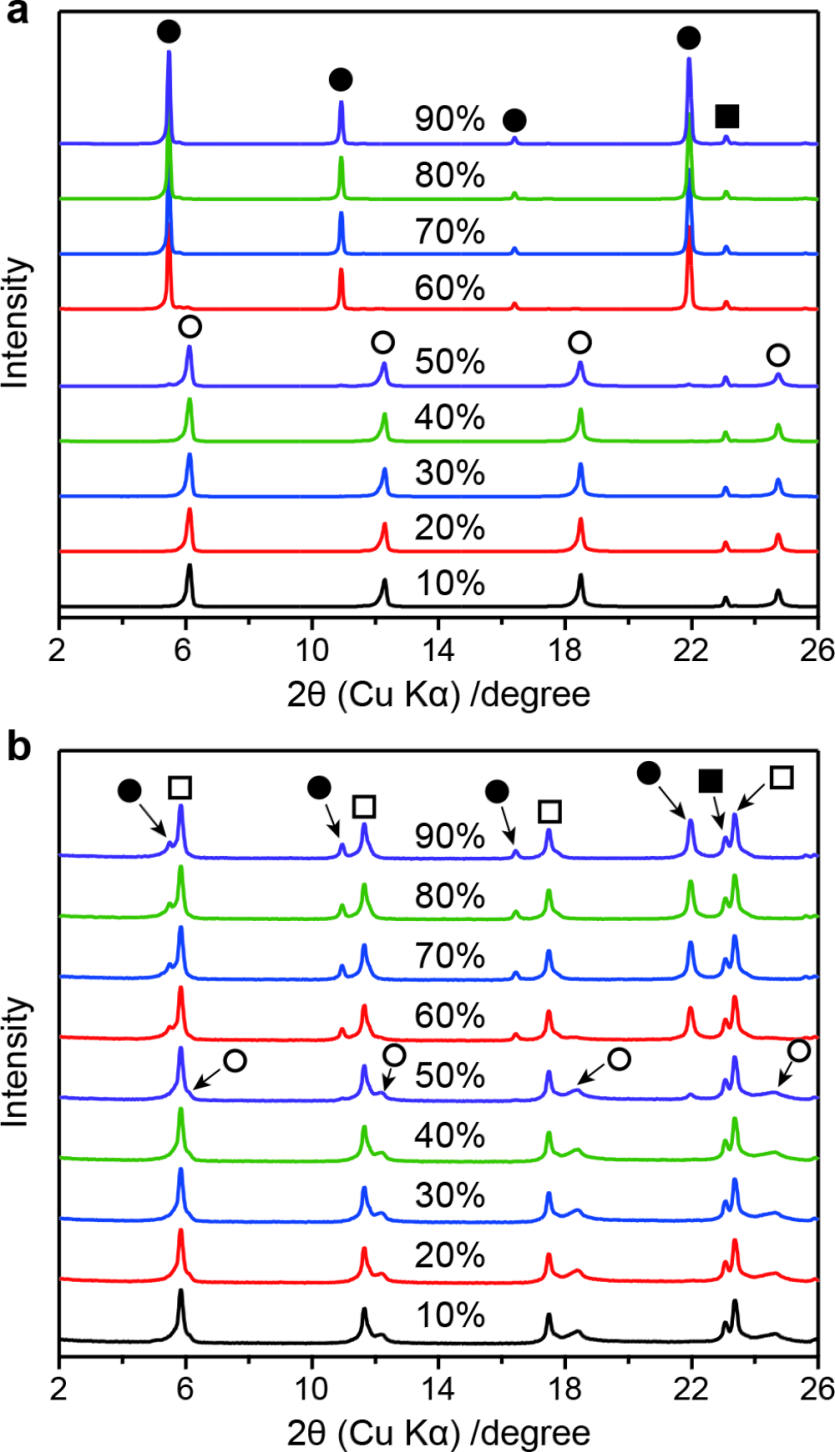
PXRD patterns of (a) fresh and (b) degraded HCa_2_Nb_3_O_10_·1.5H_2_O crystals under different relative humidities, as indicated in the figures. Peaks marked with filled circles, empty circles, and empty squares represent basal reflections corresponding to *d* spacings of 1.61, 1.44, and 1.51 nm, respectively, while those marked with filled squares indicate an in-plane 100 reflection.

The compositional changes caused by exposure to ambient air were examined in more detail by performing XRD analysis on single crystals of both fresh and degraded HCa_2_Nb_3_O_10_·1.5H_2_O. The X-ray precession pattern obtained from the (0*kl*) plane of a crystal exposed for 4 days yielded lattice constants of *b* = 0.3870(1) nm and *c* = 1.6292(6) nm (Figure 3a), which are in good agreement with the tetragonal unit cell of HCa_2_Nb_3_O_10_·1.5H_2_O.^25^ The crystal exposed to air for 18 days showed a similar pattern with nearly identical unit cell dimensions as the dominant phase (Figure 3b). However, it is noteworthy that additional diffraction spots appeared along the *c*-axis, suggesting the emergence of a new phase. The 00*l* reflections became elongated at higher *l* values and eventually split, as indicated by the arrows in the figure. These observations indicate that while the in-plane lattice plane remains unchanged between the two samples, the interlayer distance has contracted in the degraded crystal, consistent with a typical topotactic transformation. The change corresponds to an ∼6% reduction in interlayer spacing, and the resulting *d* spacing of 1.52 nm closely matches that of the nonhydratable phase observed in the PXRD pattern (Figure 2b). This supports the coexistence of two distinct phases (hydratable and nonhydratable) within a single crystal, which likely results in refractive index variations and gives rise to the “photoframe” texture seen in the degraded crystals (Figure 1d).

**Figure 3:**
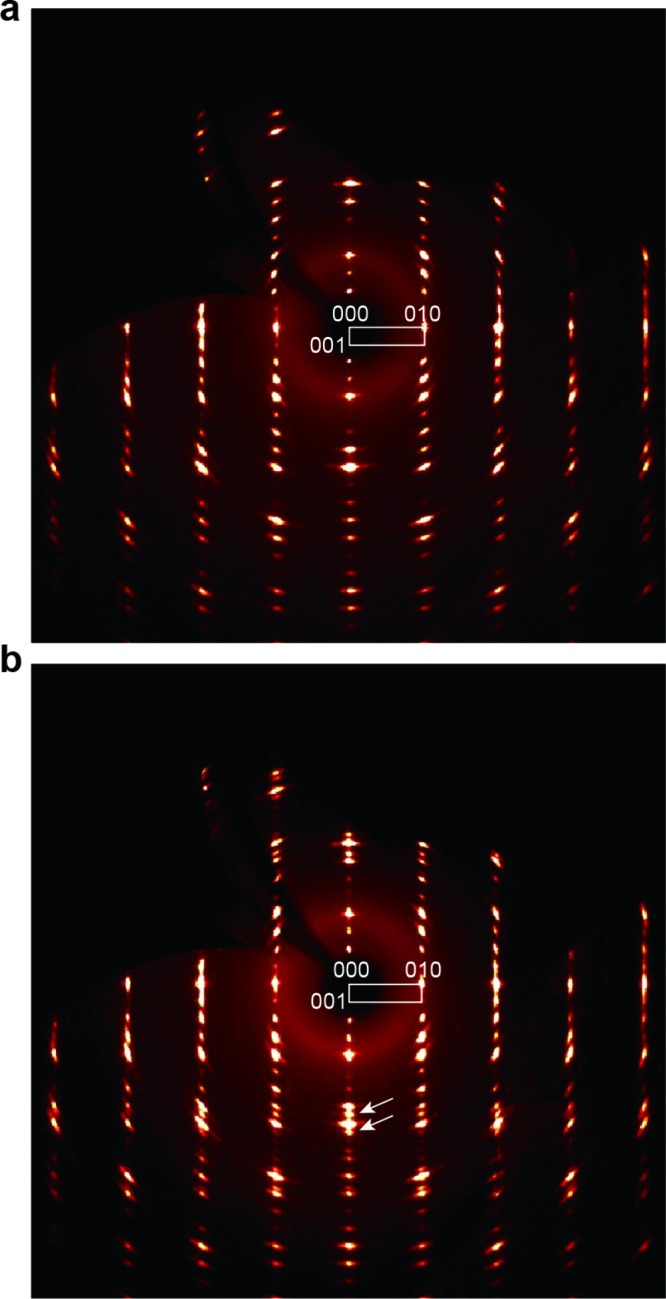
XRD precession patterns of the (0*kl*) plane of HCa_2_Nb_3_O_10_·1.5H_2_O crystals after exposure to ambient air for (a) 4 and (b) 18 days. Arrows in panel b indicate additional spots compared with panel a.

As described above, the degradation of HCa_2_Nb_3_O_10_·1.5H_2_O crystals is apparently caused by a component present in the air. In addition to major constituents such as N_2_ and O_2_, ambient air contains various minor gaseous species, including H_2_O vapor, CO_2_, noble gases, and others. Since the protonated crystals of HCa_2_Nb_3_O_10_·1.5H_2_O exhibit acidic interlayer environments, it is reasonable to consider that alkaline molecules or species in ambient air could induce chemical changes in the interlayer region. Notably, indoor air contains trace amounts of ammonia (0.02–0.12 ppm), primarily emitted from concrete walls and structural pillars in buildings.^23^ Amine-based additives, such as urea, are commonly incorporated into construction materials to lower the freezing point of water. These additives tend to decompose at elevated temperatures, releasing ammonia gas. Ammonia emitted from human activity may also contribute.^24^ Ammonia can interact with interlayer H^+^ ions in HCa_2_Nb_3_O_10_·1.5H_2_O, forming NH_4_^+^ ions. Therefore, it is most plausible that ammonia present in air plays a crucial role in the degradation of HCa_2_Nb_3_O_10_·1.5H_2_O crystals. To verify this hypothesis, fresh crystals were exposed to ammonia vapor (∼60 ppm) for 4 h and subsequently characterized by XRD and FTIR measurements. The XRD data revealed the presence of two immiscible phases with basal spacings of 1.60 and 1.52 nm (Figure 4a). The former represents the fully hydrated protonated phase, while the latter matches the nonhydratable phase observed in degraded crystals, which is also consistent with the *d* spacing of the NH_4_^+^ ion-exchanged phase.^28^ The peak corresponding to the fully hydrated protonated phase was relatively prominent in the sample exposed to ammonia vapor compared with the degraded crystals, likely due to an insufficient reaction time. FTIR analysis of the ammonia-treated crystals further supported these findings (Figure 4b). Fresh crystals exhibited vibrational bands at approximately 3360 and 1631 cm^–1^, corresponding to the stretching and bending modes of H_2_O, respectively. In contrast, both the degraded and ammonia-treated crystals showed a strong vibrational peak at 1415 cm^–1^ and broad bands in the range of 2800–3200 cm^–1^, which can be assigned to the bending and stretching modes of NH_4_^+^, respectively.^29^ Furthermore, the ammonia-treated crystals did not exhibit swelling upon contact with DMAE. Based on these results, it is confirmed that ammonia gas is responsible for the degradation of HCa_2_Nb_3_O_10_·1.5H_2_O crystals. The “photoframe” texture observed under polarized optical microscopy (Figure 1d) originates from regions where NH_3_ molecules have been intercalated to form ammonium ions. This texture is likely due to the difference in refractive index between the ammonium-intercalated and protonated regions within the crystals. These ammonium-intercalated regions show reduced solid acidity and, therefore, do not react with amines such as DMAE, which normally introduce large amounts of water into the galleries. As a result, the swelling capability is lost.

**Figure 4:**
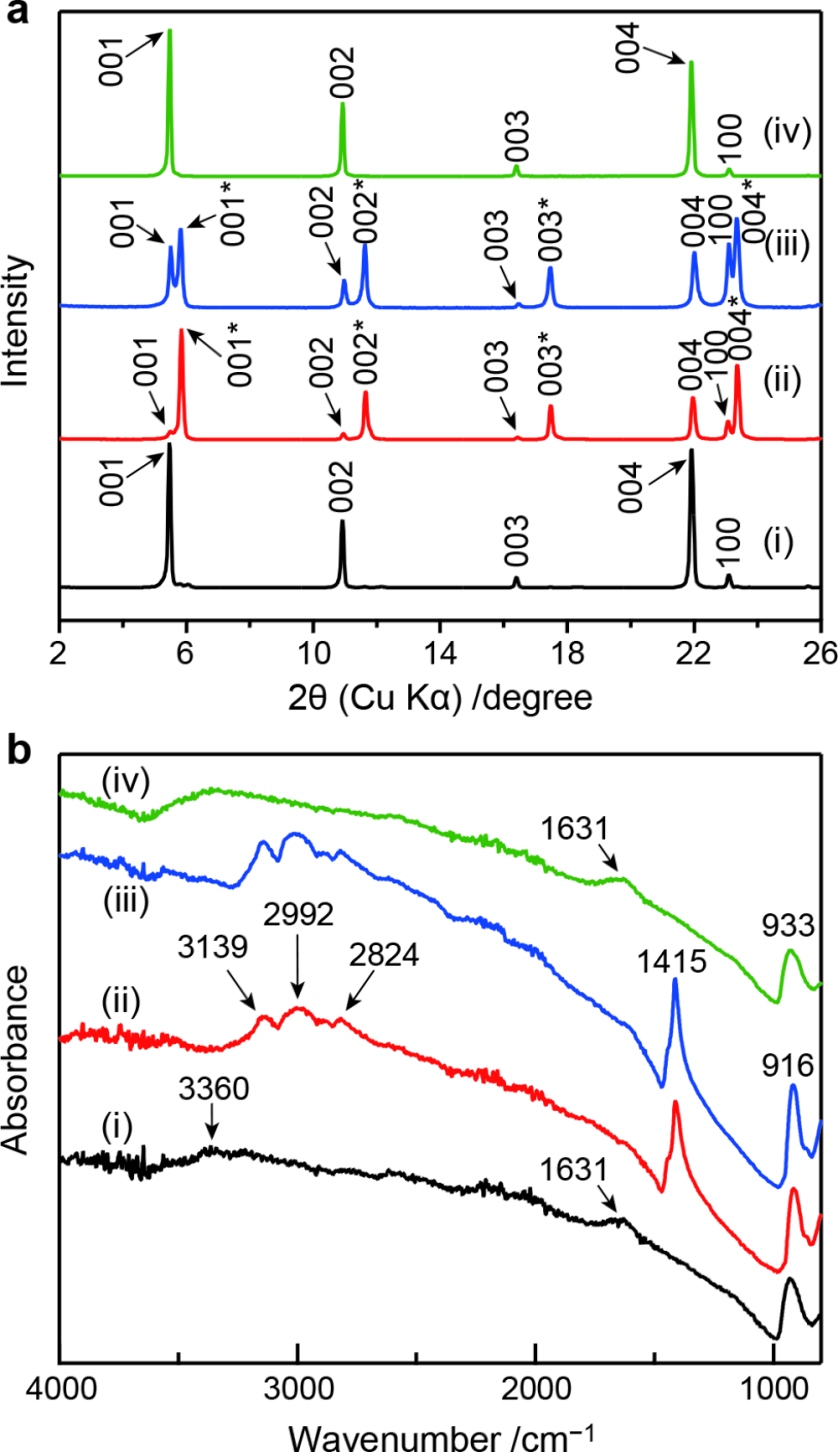
(a) PXRD patterns and (b) FTIR spectra of (i) fresh HCa_2_Nb_3_O_10_·1.5H_2_O crystals, (ii) degraded crystals after exposure to ambient air, (iii) fresh crystals after exposure to ammonia gas, and (iv) crystals subsequently treated with HNO_3_ following ammonia exposure. Indices of two different basal reflection series are indicated with and without asterisks in panel a.

Finally, we attempted to restore the swelling capability of the degraded crystals through acid treatment. The ammonia-treated crystals were dispersed in 5 mol dm^–3^ HNO_3_ for 1 day, thoroughly washed with water, and then dried in air. The recovered crystals were characterized by XRD and FTIR measurements (Figure 4). The XRD data showed a single series of sharp basal diffraction peaks corresponding to a spacing of 1.61 nm, characteristic of the fully hydrated protonated phase. Moreover, the FTIR spectrum no longer exhibited the bands attributable to NH_4_^+^, indicating that the intercalated NH_4_^+^ ions had been replaced by H^+^ according to the following reaction:

$$\;{\mathrm{NH}}_{4}{\mathrm{Ca}}_{2}{\mathrm{Nb}}_{3}O_{10}nH_{2}O+{\mathrm{HNO}}_{3}\left(\mathrm{aq}\right)\to {\mathrm{HCa}}_{2}{\mathrm{Nb}}_{3}O_{10}\cdot 1.5H_{2}O+{\mathrm{NH}}_{4}{\mathrm{NO}}_{3}\left(\mathrm{aq}\right)$$

The regenerated HCa_2_Nb_3_O_10_·1.5H_2_O crystals exhibited good swelling behavior upon immersion in the DMAE solution, demonstrating the successful recovery of swelling capability. This treatment was also effective for restoring crystals that had been degraded by prolonged exposure to ambient air over several months.

Since protonic layered metal oxides are expected to adsorb basic molecules other than ammonia and can be regenerated by acid treatment, they may serve as renewable adsorbents for various basic gases, such as methylamine. In contrast, they are not effective for the adsorption of acidic molecules, such as CO_2_.

## Conclusions

In summary, HCa_2_Nb_3_O_10_·1.5H_2_O crystals exhibited degradation of their swelling capability upon exposure to ambient air, thereby hindering their exfoliation into nanosheets. The cause of this degradation was identified as trace amounts of ammonia emitted from the surrounding environment, which was intercalated into the interlayer space by reacting with protons to form NH_4_^+^ ions. This was evidenced by a shift in the basal spacing observed in the XRD patterns and the appearance of vibrational bands corresponding to NH_4_^+^ in the FTIR spectrum. When the crystals were stored in a sealed container that suppressed extensive exposure to ammonia, their swelling capability was preserved, even after several months. Moreover, the degraded crystals could be restored through an acid treatment. Overall, this study addresses a long-standing question regarding the mechanism of degradation in layered crystals and provides a practical approach to recovering their swelling and exfoliation capabilities.

## Supplementary Material



## Notes

SEM image of HCa_2_Nb_3_O_10_·1.5H_2_O crystals and optical microscopy images of the crystals after exposure to ambient air (PDF)
