# Supplementary material for: Ammonia Uptake from Ambient Air by Protonic Layered Metal Oxide as a Cause of the Gradual Degradation of Its Swelling Reactivity
Source: Langmuir. 2025 Aug 29;41(36):24957–62. doi: 10.1021/acs.langmuir.5c03503 (PMC12444990; doi:10.1021/acs.langmuir.5c03503)
Supplement: Supplementary file 1 [file la5c03503_si_001.pdf]

Supporting Information

Ammonia Uptake from Ambient Air by Protonic Layered Metal Oxide as a Cause of the Gradual Degradation of Its Swelling Reactivity

Nobuyuki Sakai,* Ritesh Uppuluri, Nobuo Iyi, Yasuo Ebina, Renzhi Ma, and Takayoshi Sasaki

Research Center for Materials Nanoarchitectonics (MANA), National Institute for Materials Science (NIMS), 1-1 Namiki, Tsukuba, Ibaraki 305-0044, Japan

* Corresponding author. E-mail: sakai.nobuyuki@nims.go.jp

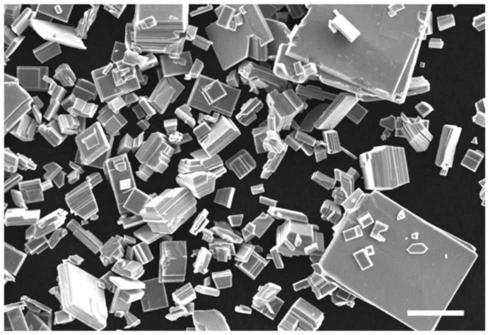

Figure S1. SEM image of $HCa_2Nb_3O_{10}\cdot 1.5H_2O$ crystals. Scale bar indicates 50 μm.

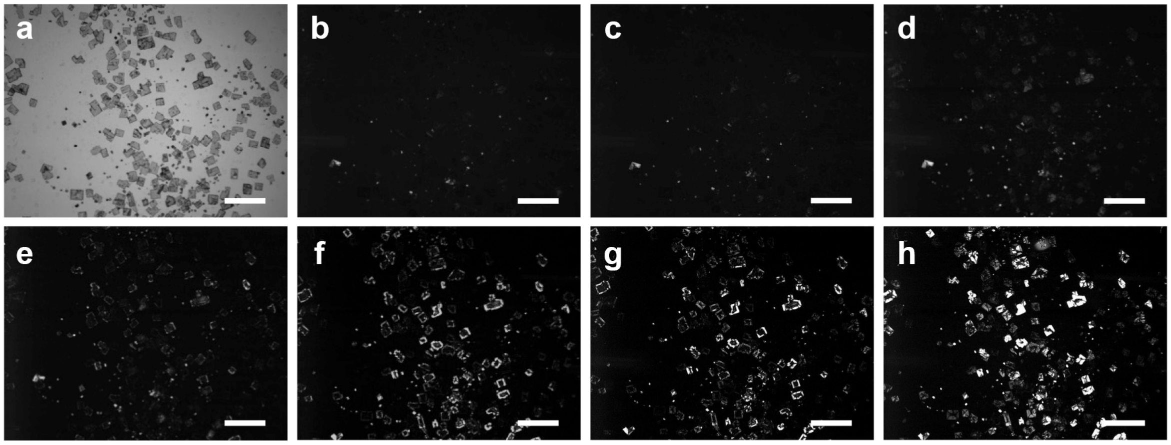

Figure S2. (a) Normal and (b-g) polarized optical microscopy images of $HCa_2Nb_3O_{10}\cdot 1.5H_2O$ crystals after exposure to ambient air for (a, b) 0, (c) 1, (d) 3, (e) 7, (f) 14, (g) 21, and (h) 49 days. Scale bars represent 500 μm.
